# Aged-Related Changes in Body Composition and Association between Body Composition with Bone Mass Density by Body Mass Index in Chinese Han Men over 50-year-old

**DOI:** 10.1371/journal.pone.0130400

**Published:** 2015-06-19

**Authors:** Ying Jiang, Ying Zhang, Mengmeng Jin, Zhaoyan Gu, Yu Pei, Ping Meng

**Affiliations:** 1 Department of Geriatric Endocrinology, PLA General Hospital, Beijing, China; 2 Department of Geriatric Nephrology, PLA General Hospital, Beijing, China; 3 Endocrinology Department, PLA General Hospital, Beijing, China; 4 Hainan Branch Health care Department, PLA General Hospital, Sanya, China; 5 Hainan Branch Endocrinology Department, PLA General Hospital, Sanya, China; 6 Health care Department, PLA General Hospital, Beijing, China; 7 Hainan Branch Nursing Department, PLA General Hospital, Sanya, China; UAMS, UNITED STATES

## Abstract

**Objectives:**

Aging, body composition, and body mass index (BMI) are important factors in bone mineral density (BMD). Although several studies have investigated the various parameters and factors that differentially influence BMD, the results have been inconsistent. Thus, the primary goal of the present study was to further characterize the relationships of aging, body composition parameters, and BMI with BMD in Chinese Han males older than 50 years.

**Methods:**

The present study was a retrospective analysis of the body composition, BMI, and BMD of 358 Chinese male outpatients between 50 and 89 years of age that were recruited from our hospital between 2009 and 2011. Qualified subjects were stratified according to age and BMI as follows: 50–59 (n = 35), 60–69 (n = 123), 70–79 (n = 93), and 80–89 (n = 107) years of age and low weight (BMI: < 20 kg/m^2^; n = 21), medium weight (20 ≤ BMI < 24 kg/m^2^; n = 118), overweight (24 ≤ BMI < 28 kg/m^2^; n = 178), and obese (BMI ≥ 28 kg/m^2^; n = 41). Dual-energy X-ray absorptiometry (DEXA) was used to assess bone mineral content (BMC), lean mass (LM), fat mass (FM), fat-free mass (FFM), lumbar spine (L1-L4) BMD, femoral neck BMD, and total hip BMD. Additionally, the FM index (FMI; FM/height^2^), LM index (LMI; LM/height^2^), FFM index (FFMI; [BMC+LM]/height^2^), percentage of BMC (%BMC; BMC/[BMC+FM+LM] × 100%), percentage of FM (%FM; FM/[BMC+FM+LM] × 100%), and percentage of LM (%LM; LM/(BMC+FM+LM) × 100%) were calculated. Osteopenia or osteoporosis was identified using the criteria and T-score of the World Health Organization.

**Results:**

Although there were no significant differences in BMI among the age groups, there was a significant decline in height and weight according to age (*p* < 0.0001 and *p* = 0.0002, respectively). The LMI and FFMI also declined with age (both *p* < 0.0001) whereas the FMI exhibited a significant increase that peaked in the 80-89-years group (*p* = 0.0145). Although the absolute values of BMC and LM declined with age (*p* = 0.0031 and *p* < 0.0001, respectively), there was no significant difference in FM. In terms of body composition, there were no significant differences in %BMC but there was an increase in %FM (*p* < 0.0001) and a decrease in %LM (*p* < 0.0001) with age. The femoral neck and total hip BMD significantly declined with age (*p* < 0.0001 and *p* = 0.0027, respectively) but there were no differences in L1-L4. BMD increased at all sites (all *p* < 0.01) as BMI increased but there were declines in the detection rates of osteoporosis and osteopenia (both *p* < 0.001). A logistic regression revealed that when the medium weight group was given a BMI value of 1, a decline in BMI was an independent risk factor of osteoporosis or osteopenia, while an increase in BMI was a protective factor for BMD. At the same time, BMD in L1-L4 exhibited a significant positive association with FMI (*p* = 0.0003) and the femoral neck and total hip BMDs had significant positive associations with FFMI and LMI, respectively (both *p* < 0.0001).

**Conclusions:**

These data indicate that LMI and FFMI exhibited significant negative associations with aging in Chinese Han males older than 50 years, whereas FMI had a positive association. BMD in the femoral neck and total hip declined with age but an increased BMI was protective for BMD. LMI and FFMI were protective for BMD in the femoral neck and total hip.

## Introduction

Because the average life expectancy has increased in recent years, the presence of health problems in the elderly has become a prevalent issue. Osteoporosis and osteopenia, or a decline in bone mineral density (BMD) that leads to an increased risk of fracture, are both causes of mortality and disability in elderly adults and represent an enormous cost to health care [1].

Many factors influence BMD. For example, body weight is an important determinant of bone mass [2, 3] and should be regarded as an important risk factor of osteoporosis and osteopenia [4]. Body weight is composed of three main components: lean mass (LM), fat mass (FM) and bone. Traditionally, body composition is determined by the relative values of lean mass (LM), fat mass (FM), fat-free mass (FFM), and bone mineral content (BMC) and it is known that each of these factors changes with age. As a result, many studies have investigated the relationship between aging and body composition parameters and the role of these parameters on BMD. A large-scale Western European study[5] involving subjects between 15 and 98 years of age found that FM increases with age in both sexes, whereas LM declines. In contrast, another study reported that, while LM decreases with age, there were no significant changes in FM in healthy Korean subjects over the age of 50 years [6]. It is possible that these discrepant findings may be due to ethnic or cultural differences; however, no study to date has investigated age-related changes in the body composition parameters of Chinese Han males over the age of 50 years.

The nature of the relationships of body composition parameters with variations in BMD has been highly contentious. Moreover, few studies have investigated the BMDs of different skeleton sites according to body mass index (BMI) in males. Several studies have demonstrated that both FM and LM contribute to the determination of bone mass [7, 8] while others have found that LM has a greater impact on BMD and may protect against the risk of fractures [9–12]. On the other hand, other studies have suggested that FM, rather than LM, is the most important determining factor for BMD [13–15] and that FM might positively contribute to BMD only in older males [16]. The discrepant findings of these studies suggest that BMD is dependent on a variety of factors including gender, ethnicity, BMI, and age.

Previous studies evaluating the preservation of BMD have found that BMI is an important indicator of bone tissue structure [17–19] and that it is related to the risk of osteoporotic fractures [20, 21]. Moreover, BMI is one of the least complicated and most frequently used indicators of health that is significantly related to BMD [22]. Relative to a BMI of 25 kg/m^2^, a BMI of 20 kg/m^2^ is associated with a nearly twofold increase in the risk ratio of hip fracture [23] while a BMI of 30 kg/m^2^ is associated with an only 17% reduction in the risk of hip fracture. Therefore, a low BMI confers a substantial risk for all fractures that is largely independent of age and sex but dependent on BMD.

To better characterize this risk, the present study investigated age-related changes in body composition and BMD and the relationship between BMI and BMD in Chinese Han males over 50 years of age. Additionally, the relative contributions of various body composition parameters to BMD at different skeletal sites were evaluated based on BMI. This study aimed to provide new information regarding potential pharmaceutical targets for the development of osteoporosis therapies.

## Materials and Methods

### Subjects

The present study initially recruited and evaluated 392 Chinese male outpatients over 50-year-old who had undergone routine physical check-ups and dual energy X-ray absorptiometry (DEXA) in our hospital from 2009–2011. Exclusion criteria were history of metabolic bone diseases such as chronic liver or renal failure, hyperthyroidism and rheumatoid arthritis; history of diseases affecting body weights or composition such as thyrotoxicosis, hypothyroidism; the presence of major debilitating disease; major cardiovascular events; none of the subjects had primary or secondary low levels of gonadal hormones or had treated with medicine capable of influencing BMD, weight and body composition such as thyroid hormones, glucocorticosteroids, bisphosphonates and anti-obesity drugs within the previous 3 months. In the end, 358 men were included in the analysis and stratified into four age groups: 50–59 (n = 35), 60–69 (n = 123), 70–79 (n = 93), and 80–89 (n = 107) years of age. An experienced operator collected the measurements of body composition parameters, BMI, and BMD.

### Ethics Statement

The present study was conducted with the approval of the Ethics Committee of the Chinese PLA General Hospital (Beijing, People`s Public of China). The investigators complied with all applicable regulatory and legal requirements and the Declaration of Helsinki from 1975 (as revised in 1983). Prior to inclusion in the study, each subject provided written informed consent and none of the subjects were involved in any study-related activity without giving appropriate written informed consent. Subject confidentiality was strictly maintained throughout the study.

### Anthropometric Measurements

A variety of anthropometric measurements, including weight and height, were measured while the subjects were in light clothing without shoes. Body weight was measured to the nearest 0.1 kg and body height was measured with a hypsometer to the nearest 0.1 cm; both values were recorded as the mean of three measures. BMI was calculated as follows: [weight (kg)/height (m^2^)]. In China, BMI was divided into three levels as normal weight (18.5≤BMI< 24 kg/m2), overweight (24≤BMI< 28 kg/m2), and obesity (BMI≥28 kg/m2) [24–26]. According to our original data, there was no patients`BMI less than 18.5kg/m2. In western countries [23], there was a comparison of BMI ≥25 kg/m2 and <20 kg/m2 in hip fracture. Subsequently, we divided BMI into four groups, low weight (BMI< 20 kg/m2;n = 21), medium weight (20 ≤ BMI < 24 kg/m2; n = 118), overweight (24 ≤ BMI < 28 kg/m2; n = 178) and obese (BMI ≥ 28 kg/m2; n = 41) [27].

### BMD and Body Composition Measurements

All subjects had undergone dual-energy x-ray absorptiometry (DXA) scan (GE Lunar Prodigy Advance; GE Healthcare, WI, USA), with an in vivo precision (% coefficient of variation) of <1% for anterior–posterior spinal, femoral, total body BMD and body composition, which is increasingly used for a variety of clinical and research applications to assess BMD and body composition.

BMD (g/cm2) was measured in the lumbar spine (L1-L4), femoral neck, and total hip.[28,29] The accurate and precise values of these body composition parameters were also estimated from the DXA scan of the total body, which included BMC, LM, FM, and FFM. Additionally, the FM index (FMI; FM/height2), LM index (LMI; LM/height2), FFM index (FFMI; [BMC+LM]/height2), percentage of BMC (%BMC; BMC/[BMC+FM+LM] × 100%), percentage of FM (%FM; FM/[BMC+FM+LM] × 100%), and percentage of LM (%LM; LM/(BMC+FM+LM) × 100%) were calculated. All scans were acquired and analyzed by the same experienced operator, adhering to the guidelines provided by the manufacturer.

### Osteopenia or Osteoporosis Diagnoses

The diagnoses of osteopenia and osteoporosis were made using the T-score criteria of the World Health Organization (WHO; -2.5 < T-score < -1 and T-score ≤ -2.5, respectively). If a subject had a low T-score based on the BMD of L1-L4, femoral neck, or total hip, then that subject was classified as having osteoporosis or osteopenia.

### Statistical Analysis

All data were entered using Microsoft Excel 2010 for Windows and analyzed with SPSS version 19.0 (IBM Corporation; Armonk, New York). The data are presented as the means ± standard deviations (SD) for continuous variables and as frequencies for categorical variables. For the continuous variables, a one-way analysis of variance (ANOVA) was used to compare the associations among the anthropometric data, body composition parameters, and absolute values of the BMDs at different sites among the different age groups. The absolute values of the BMDs in the different BMI groups and the associations among the body composition parameters and the BMDs at different sites were also assessed by ANOVA. Chi-squared tests were used to compare the detection rates of osteoporosis or osteopenia among the BMI groups and an ordinal logistic regression was used to determine whether there were linear relationships among the detection rates of osteoporosis or osteopenia and the BMI groups. A p value < 0.05 was considered to indicate statistical significance.

## Results

### Subject Characteristics

**Fig 1:**
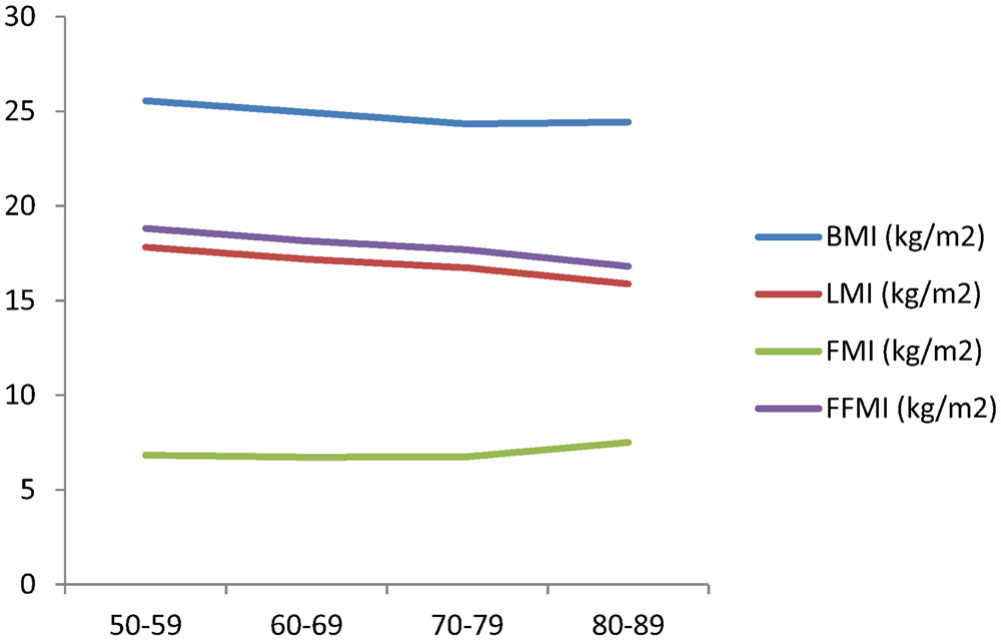
Trend of BMI, LMI, FMI, FFMI with aging.

The basic characteristics of the subjects are described in Table 1. Although there were no significant differences in BMI among the age groups, there was a significant decline in height and weight according to age (*p* < 0.0001 and *p* = 0.0002, respectively). The absolute values for BMC and LM declined with age (*p* = 0.0031 and *p* < 0.0001, respectively) but there were no significant differences in FM (*p* = 0.0704). Table 1 also shows the changes in BMI, LMI, FMI, and FFMI according to age. The LMI and FFMI exhibited significant declines with age (both *p* < 0.0001) but there was a significant increase in FMI in the 80–89-years age group (*p* = 0.0145). These changes are also depicted in graphical form (Fig 1, S1 Table).

**Table 1:** Subject characteristics by aging.

Variables	Total	Group1	Group2	Group3	Group4	*p*-value
	Mean±SD	50–59ys	60–69ys	70–79ys	80–89ys	
	(Min-Max)	Mean±SD	Mean±SD	Mean±SD	Mean±SD	
N	358	35	123	93	107	
Age(years)	72.80±9.46	56.99±2.12^b^ ^c^ ^d^	65.82±2.90^a^ ^c^ ^d^	74.72±2.82^a^ ^b^ ^d^	84.32±2.63^a^ ^b^ ^c^	< .0001
	51.80;89.60					
Height (cm)	170.40±5.11	173.17±5.40^b^ ^c^ ^d^	171.3±4.78^a^ ^c^ ^d^	169.7±5.01^a^ ^b^	168.9±4.94^a^ ^b^	< .0001
	156.00;186.00					
Weight (Kg)	71.82±9.18	76.68±9.29^b^ ^c^ ^d^	73.30±8.97^a^ ^c^ ^d^	70.26±9.35^a^ ^b^	69.86±8.44^a^ ^b^	0.0002
	49.00;108.00					
BMI (kg/m2)	224.71±2.72	25.56±2.79	24.96±2.63	24.34±2.83	24.45±2.64	0.0679
	16.54;34.84					
Body composition measures						
LMI (kg/m2)	16.75±1.41	17.83±1.55^b^ ^c^ ^d^	17.20±1.27^a^ ^c^ ^d^	16.74±1.19^a^ ^b^ ^d^	15.89±1.23^a^ ^b^ ^c^	< .0001
	13.29;21.09					
FMI (kg/m2)	6.97±2.00	6.83±1.72	6.73±1.78^d^	6.75±2.22^d^	7.50±2.06^b^ ^c^	0.0145
	1.21;14.04					
FFMI (kg/m2)	17.70±1.46	18.82±1.61^b^ ^c^ ^d^	18.16±1.32^a^ ^c^ ^d^	17.69±1.22^a^ ^b^ ^d^	16.82±1.27^a^ ^b^ ^c^	< .0001
	14.05;22.09					
BMC (g)	2763.53±397.55	2990.21±	2796.89±	2742.82±	2669.05±	0.0031
	1490.51;4010.02	420.64^b^ ^c^ ^d^	361.10^a^ ^d^	348.44^a^	438.68^a^ ^b^	
FM (g)	20267.49±6001.96	20484.06±	19765.66±	19501.84±	21439.01±	0.0704
	3412.34;41224.32	5217.34	5523.05	6569.79	6149.49	
LM (g)	48684.51±5144.72	53475.68±	50513.94±	48268.25±	45376.11±	< .0001
	35427.33;64000.20	5348.96^b^ ^c^ ^d^	4630.97^a^ ^c^ ^d^	4356.19^a^ ^b^ ^d^	4018.53^a^ ^b^ ^c^	
%BMC	3.87±0.45	3.90±0.45	3.84±0.37	3.92±0.46	3.85±0.53	0.5447
	2.62;5.95					
%FM	27.83±5.83	26.30±4.96^d^	26.67±4.76^d^	27.02±6.50^d^	30.35±5.89^a^ ^b^ ^c^	< .0001
	6.41;44.25					
%LM	68.30±5.70	69.80±4.81^d^	69.49±4.66^d^	69.06±6.31^d^	65.79±5.78^a^ ^b^ ^c^	< .0001
	52.01;88.69					
BMD measures (g/cm2)						
L1-4	1.22±0.20	1.17±0.19	1.19±0.16	1.25±0.20	1.24±0.23	0.0943
	0.81;2.12					
FN	0.88±0.14	0.97±0.14^b^ ^c^ ^d^	0.91±0.12^a^ ^d^	0.89±0.12^a^ ^d^	0.83±0.15^a^ ^b^ ^c^	< .0001
	0.54;1.45					
TH	0.99±0.14	1.04±0.16^d^	1.00±0.12^d^	0.99±0.12^d^	0.94±0.17^a^ ^b^ ^c^	0.0027
	0.58;1.47					

Abbreviations: N = number, BMI = body mass index, FMI = fat mass index, LMI = lean mass index, FFMI = fat free mass index, BMC = bone mineral content, FM = fat mass, LM = lean mass, BMD = bone mineral density, L1-4 = lumbar spine1-4, FN = femoral neck, TH = total hip, SD = standard deviation.

*p*-value is the analysis of variance between groups.

^a^ is Group 2,3,4 VS Group1, *p*<0.05.

^b^ is Group 1,3,4 VS Group2, *p*<0.05.

^c^ is Group 1,2,4 VS Group3, *p*<0.05.

^d^ is Group 1,2,3 VS Group4, *p*<0.05.

**Fig 2:**
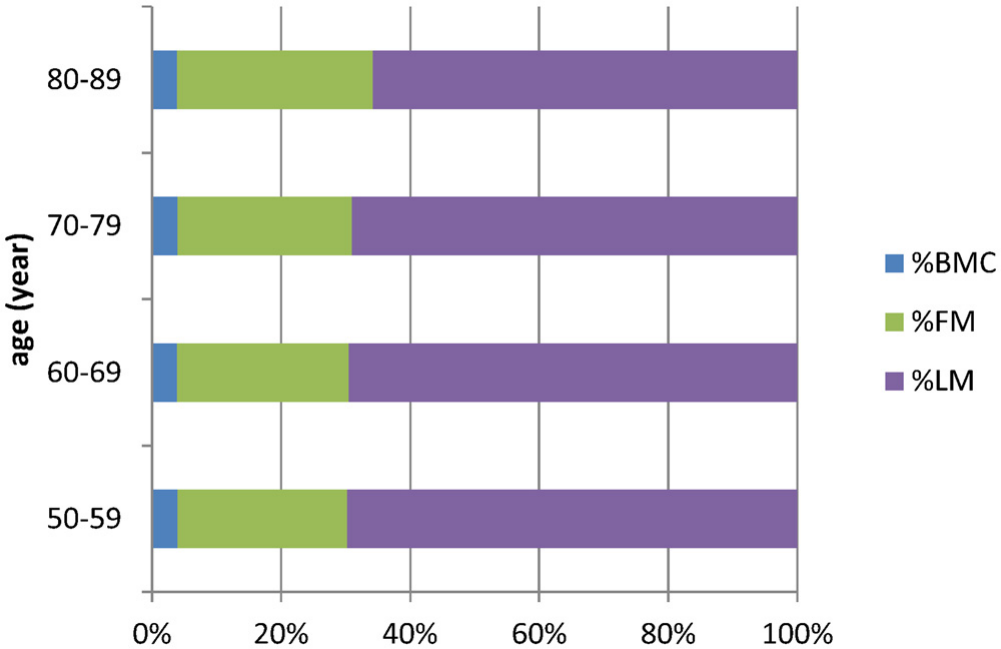
The proportion of body composition change in different age group.

**Fig 3:**
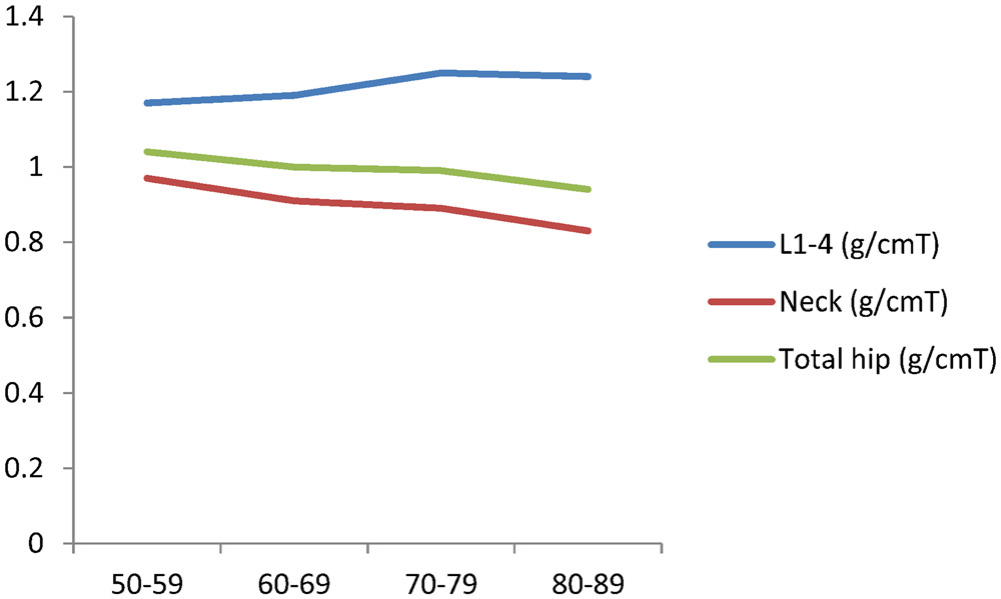
Trend of BMD at different site with aging.

According to the body composition data, there were no significant differences in %BMC among the age groups (*p* = 0.5447), but there was an increase in %FM (*p* < 0.0001) and a decrease in %LM (*p* < 0.0001; Table 1). Similarly, there was a significant increase in %FM and a significant decrease in %LM in the 80–89-years age group (Fig 2, S2 Table). BMD in the femoral neck and total hip exhibited significant declines with age (*p* < 0.0001 and *p* = 0.0027, respectively) but there was no difference in BMD at L1-L4 (*p* = 0.0943; Table 1, Fig 3, S3 Table).

### BMI and BMD Analyses

To investigate the interaction between BMI and BMD, the subjects were categorized into four groups based on BMI. The absolute values of the BMDs at each of the different sites showed significant positive associations with BMI (all *p* < 0.01; Table 2). Additionally, there was a positive correlation between BMI and the absolute values of the BMDs such that they increased in conjunction with each other. The detection rates of osteoporosis and osteopenia had significant negative associations with BMI but osteoporosis was not correlated with BMD at L1-L4 (all *p* < 0.001; Table 3).

**Table 2:** The change of absolute value in BMD at different site by different BMI level.

Variables	BMI (Kg/m2)				
	Group 1	Group 2	Group 3	Group 4	*p*-value
	<20	≥20<24	≥24<28	≥28	
N	21	118	178	41	
L1-4	1.09±0.21^b^ ^c^ ^d^	1.20±0.19^a^ ^c^	1.24±0.20^a^ ^b^	1.25±0.17^a^	0.0038
FN	0.80±0.17^b^ ^c^ ^d^	0.87±0.13^a^ ^d^	0.89±0.13^a^	0.94±0.14^a^ ^b^	0.0011
TH	0.86±0.17^b^ ^c^ ^d^	0.96±0.14^a^ ^c^ ^d^	1.00±0.13^a^ ^b^ ^d^	1.05±0.14^a^ ^b^ ^c^	<0.001

Abbreviations: N = number, BMI = body mass index, L1-4 = lumbar spine1-4, FN = femoral neck, TH = total hip.

*p*-value is the analysis of variance between groups.

^a^ is Group 2,3,4 VS Group1, *p*<0.05.

^b^ is Group 1,3,4 VS Group2, *p*<0.05.

^c^ is Group 1,2,4 VS Group3, *p*<0.05.

^d^ is Group 1,2,3 VS Group4, *p*<0.05.

**Table 3:** The detection rate of osteoporosis or osteopenia by different BMI level.

Variables	BMI (Kg/m2)				
	<20	≥20<24	≥24<28	≥28	*p*-value
N	21	118	178	41	
L1-4					
Normal	12 (57.14)	106 (89.83)	166 (93.26)	40(97.56)	< .0001
Osteopenia	9 (42.86)	12 (10.17)	12 (6.74)	1 (2.44)	
Osteoporosis	0	0	0	0	
FN					
Normal	8 (38.10)	62 (52.54)	121 (67.98)	29 (70.73)	0.0009
Osteopenia	9 (42.86)	51 (43.22)	50 (28.09)	11 (26.83)	
Osteoporosis	4 (19.05)	5 (4.24)	7 (3.93)	1 (2.44)	
TH					
Normal	12 (57.14)	92 (77.97)	153 (85.96)	37 (92.50)	0.0034
Osteopenia	7 (33.33)	24 (20.34)	23 (12.92)	4 (9.76)	
Osteoporosis	2(9.52)	2 (1.69)	2 (1.12)	0 (0)	
Total					
Normal	6 (28.57)	60 (50.85)	120 (67.42)	30 (73.17)	0.0002
Osteopenia	11 (52.38)	53 (44.92)	52 (29.21)	10 (25.00)	
Osteoporosis	4 (19.05)	5 (4.24)	6 (3.37)	1 (2.50)	

Abbreviations: N = number, BMI = body mass index, L1-4 = lumbar spine1-4, FN = femoral neck, TH = total hip.

Total means any of the three sites detected for osteoporosis or osteopenia that is considered as osteoporosis or osteopenia.

*p*-value is the result of chi-square test.

### Risk Factors

A logistic regression model was used to evaluate the odds ratios (ORs) and 95% confidence intervals (CIs) of having osteoporosis or osteopenia for each BMI group compared with the highest group with adjustments for age. The risk of having osteoporosis or osteopenia progressively increased from the highest BMI group to the lowest BMI group. After adjusting for age, the multivariable adjusted ORs for osteoporosis or osteopenia in the lowest BMI group and the highest BMI group were 2.69 (95% CI: 1.06–6.83, *p* = 0.038) and 0.45 (95% CI: 0.20–0.99, *p* = 0.046) compared to the normal BMI group (Table 4).

**Table 4:** Comparison of the detection rate of osteopenia or osteoporosis in different BMI group.

BMI	Unadjusted-OR		Adjusted-OR*	
(Kg/m2)	OR (95%CI)	*p*-value	Adj.OR (95%CI)	*p*-value
<20	3.105(1.233–7.818)	0.0162	2.688(1.057–6.833)	0.0378
≥20<24	1		1	
≥24<28	0.513(0.320–0.823)	0.0057	0.461(0.283–0.751)	0.0019
≥28	0.405(0.186–0.879)	0.0224	0.445(0.201–0.986)	0.0461

Abbreviations: BMI = body mass index, OR = odd ratio, Adj.OR = adjusted odd ratio, CI = confidence interval, Adjusted-OR

*: adjusted for age.

### Analysis of body composition parameters and BMD

Table 5 shows the changes in BMD at different sites based on FMI, LMI, and FFMI quartiles. According to the FMI quartiles, there were no significant differences in BMD in the femoral neck (*p* = 0.3981) but the BMDs in L1-L4 were significantly higher as the FMI quartiles increased (*p* = 0.0003). BMD in the femoral neck and total hip also significantly increased as the LMI and FFMI increased (all *p* < 0.0001).

**Table 5:** Comparison the change of BMD at different site based on the quartile of FMI, LMI and FFMI.

Variables		Group 1	Group 2	Group 3	Group 4	*p*-value
		Q1(N = 89)	Q2(N = 90)	Q3(N = 89)	Q4(N = 90)	
FMI						
L1-4	Mean±SD	1.16±0.20^c^ ^d^	1.20±0.17^d^	1.25±0.20^a^	1.27±0.20^a^ ^b^	0.0003
	Median(Q1-Q4)	1.10(1.02–1.28)	1.19(1.07–1.34)	1.22(1.11–1.37)	1.26(1.14–1.34)	
FN	Mean±SD	0.88±0.15	0.87±0.12	0.89±0.15	0.90±0.13	0.3981
	Median(Q1-Q4)	0.88(0.79–0.96)	0.86(0.80–0.93)	0.90(0.80–0.97)	0.90(0.82–0.98)	
TH	Mean±SD	0.97±0.15^d^	0.96±0.13 ^d^	0.99±0.15	1.02±0.13^a^ ^b^	0.0252
	Median(Q1-Q4)	0.97(0.87–1.06)	0.97(0.89–1.04)	1.01(0.90–1.09)	1.03(0.93–1.09)	
LMI						
L1-4	Mean±SD	1.22±0.22	1.21±0.21	1.24±0.19	1.21±0.16	0.8275
	Median(Q1-Q4)	1.20(1.06–1.35)	1.20(1.06–1.33)	1.21(1.08–1.36)	1.21(1.09–1.32)	
FN	Mean±SD	0.84±0.15^c^ ^d^	0.86±0.12 ^c^ ^d^	0.91±0.13^a^ ^b^	0.93±0.13^a^ ^b^	< .0001
	Median(Q1-Q4)	0.83(0.74–0.93)	0.86(0.77–0.95)	0.91(0.82–1.00)	0.91(0.85–0.98)	
TH	Mean±SD	0.93±0.15^c^ ^d^	0.96±0.13 ^c^ ^d^	1.01±0.13 ^a^ ^b^	1.04±0.13 ^a^ ^b^	< .0001
	Median(Q1-Q4)	0.95(0.84–1.03)	0.96(0.87–1.03)	1.02(0.92–1.10)	1.02(0.96–1.11)	
FFMI						
L1-4	Mean±SD	1.21±0.22	1.22±0.20	1.23±0.20	1.23±0.16	0.7502
	Median(Q1-Q4)	1.19(1.06–1.34)	1.20(1.07–1.32)	1.20(1.07–1.35)	1.22(1.11–1.33)	
FN	Mean±SD	0.84±0.15^c^ ^d^	0.86±0.12 ^c^ ^d^	0.90±0.13^a^ ^b^	0.94±0.13 ^a^ ^b^	< .0001
	Median(Q1-Q4)	0.83(0.73–0.93)	0.86(0.78–0.94)	0.90(0.81–0.99)	0.92(0.86–1.00)	
TH	Mean±SD	0.93±0.16 ^c^ ^d^	0.96±0.13 ^c^ ^d^	1.01±0.13 ^a^ ^b^	1.04±0.12 ^a^ ^b^	< .0001
	Median(Q1-Q4)	0.93(0.83–1.03)	0.97(0.88–1.03)	1.01(0.91–1.09)	1.04(0.97–1.12)	

Abbreviations: N = number, BMI = body mass index, L1-4 = lumbar spine1-4, FN = femoral neck, TH = total hip.

*p*-value is the analysis of variance between groups.

^a^ is Group 2,3,4 VS Group1, *p*<0.05.

^b^ is Group 1,3,4 VS Group2, *p*<0.05.

^c^ is Group 1,2,4 VS Group3, *p*<0.05.

^d^ is Group 1,2,3 VS Group4, *p*<0.05.

## Discussion

To our knowledge, the present study is the first to investigate age-related changes in body composition and BMD in Chinese Han males over 50 years of age. Additionally, the present study evaluated the relationship between BMI and BMD and the relative contributions of various body composition parameters to BMD at different skeletal sites according to BMI. The findings demonstrated that LM, LMI, %LM and BMC diminished with age, which is consistent with previous reports [27]. FM, another important body composition parameter, did not exhibit age-related changes but there was a tendency to increase in the 80–89-years age group. Moreover, FMI and %FM peaked in the 80–89-years age group.

As age increased, there was a decline in body weight that was reflected in the reduced values of the body composition parameters. There are several factors influencing the body composition. Previous studies [4,30–33] indicated that aging and estrogen levels of menopause are two important ones in postmenopausal women. Serum levels of sclerostin [34] may also play a key role. In men, except aging, sex steroid hormone levels [35,36] may have association with the parameters of body composition, but the results are still equivocal.

There are several ways for assessing body composition, such as anthropometry, bioelectrical impedance analysis (BIA), magnetic resonance imaging (MRI) and computed tomography (CT), dual-energy X-ray absorptiometry (DXA)[37–42]. BIA is a kind of screening method which is lack of accuracy. CT and MRI are more accurate in assessing muscle and fat areas in cadaveric studies [43,44]. However, they are expensive, time-consuming and/or require radiation, and have limited availability. DXA has relative availability, inexpensive, and low radiation dose. Studies also have shown strong correlations between body composition parameters obtained by DXA and by CT or MRI in adults. [38–40,45–47]. For the past two decades, DXA have been characterized for a time-efficient and minimal-risk method of assessing both BMD and body composition.

FM accounts for approximately 16% of total body weight in normal-weight males, and %FM tends to increase until approximately 90 years of age [48]. Several studies [5, 49, 50] have observed that FM increases after 74 years of age and %FM increased throughout the lifetime of males. The present findings support these results but neither the present nor previous studies could confirm a highly significant curvilinear relationship between age and FM. It has been noted that FM peaks in late middle age [51] and, therefore, it appears that age-related changes in body composition are complicated and need to be confirmed by longitudinal studies.

LM decreases after 60 years of age and is associated with body weight [52, 53]. In the our study, LM was highest in the age 50–59-years but declined thereafter, probably because the weight gains of the subjects were no longer sufficient to offset the inevitable loss of LM that occurs with aging. The reduction rates of BMC and LM might exceed the rate of FM increasing, which may be the reason for the decreased body weight. The tendencies of BMC, LM, and FM to decrease with aging in Chinese Han elderly males may be etiological factors associated with the development of other diseases, such as age-related losses of muscle mass or sarcopenia.

Human bone tissue begins to decline at approximately 40 years of age due to the dysfunction of osteoblasts and a relative increase in the reabsorption of osteoclasts, which results in the decrease of bone mass. In the present study, BMD had a negative association with BMI and higher levels of obesity decreased the risk of osteoporosis or osteopenia. Males with a BMI < 20 had a 6.04-fold higher age-adjusted risk of osteoporosis than those with a BMI ≥ 28, which is similar to the previous study[54]. A hospital-based study of elderly males suggested that overweight and obese males are more likely to have osteoporosis and osteopenia [55] while another study found that a lower BMI is associated with lower BMD [56]. The mechanisms whereby adipose tissue exerts positive effects on BMD remain unclear. However, it is possible that the association of obesity with BMD is based in the conversion of androgen to estrogen [57], which improves bone mass in both males and females [58, 59] and maintains healthy plasma levels of insulin and regulating factors such as insulin-like growth factor-1, leptin, and adiponectin [60]. Additionally, obesity can also provide cushioning for the hip in the event of a fall [20].

The effects of body composition on BMD are well known, but whether FM or LM has a greater influence on BMD remains controversial. Some studies have found that total FM is not related to bone mineral measures in males [61–63], while others have shown different results[14,15, 64–65]. Moreover, LM is the only independent factor which contributes to BMD in the lumbar, femoral neck, and total hip sites in older males [62] but it is also shown that the influence of LM on femoral neck is greater than that on lumbar spine [66]. The discrepancy is due to the differences in race, the measured sites and age. Therefore, it is necessary to assess the independent effects of body composition parameters on BMD at different sites to develop strategy for the prevention of osteoporosis or osteopenia in elderly Chinese males. In present study, we found that FMI had a positive association with BMD at L1-L4, whereas LMI and FFMI had significant positive associations with BMD in the femoral neck and total hip.

A previous study of 1000 males between 71 and 90 years old indicated that the radiographic features of lumbar disc degeneration, anterior osteophytes, and end-plate sclerosis were associated with BMD increasing in the lumbar spine [67]. However, BMD in the femoral neck and total hip are considerably less affected by other factors [68]. The present study also demonstrated that BMD in the femoral neck and total hip were significantly influenced by aging. But there was no change in BMD in the lumbar spine, which has been reported [28]. Therefore, it may be concluded that LMI and FFMI had a stronger relationship with BMD than FMI in elderly males.

As an important component of body weight, skeletal muscle contributes to LM and FFM plays an important role in bone biomechanics. As a complete unit of motor function, bone and skeletal muscle are closely linked. The stress generated by muscle contractions can lead to bone-specific deformations of bone tissue which, in turn, stimulates bone cells and osteoblasts, increases the synthesis and expression of osteoblast-related genes, and improves bone level so that the skeleton can adapt to applied pressure. A lesser degree of LM or FFM can account for a lack of physical exercise and, in fact, there are significant associations among a lack of physical exercise and low bone mass. Muscle, a primary contributing factor to LM and FFM, contracts to produce mechanical strength and regulates variations in BMD. Muscle contractions are important factors that contribute to increased bone mass and which can produce mechanical stress, which irritates osteoblasts and increases bone formation. LM or FFM can vary, therefore, the adoption of a healthy lifestyle that includes movement and exercise should be encouraged so as to increase mechanical load and enhance lean body mass. Ultimately, this will increase BMC and reduce the risk of osteoporosis.

In summary, the present study revealed an age-associated decline in BMD in the femoral neck and total hip of elderly males. Furthermore, the present findings suggest that LM and FFM may be important determinants of BMD in the femoral neck and total hip. Based on these data, it appears that lifestyle factors, including physical activity, that benefit bone health and LM or FFM should be encouraged to prevent diseases such as osteoporosis or sarcopenia.

Our study has some limitations. Firstly, as a cross-sectional study, though had enough samples, the present analysis is limited in its ability to elucidate causal relationships between aging, body composition parameters and BMD. Secondly BMD does not represent the entirety of bone mass, thus, further study is required to elucidate the relationships among body composition parameters and the risk of fracture. Thirdly, DXA has relative availability, inexpensive, and low radiation dose. But DXA is still not the golden stander in measuring body composition. It may have some deviation. Fourthly, the study cohort has the limitation of region and ethnicity.

## Supporting Information

S1 Table The data of Body mass index(BMI), lean mass index(LMI), fat mass index(FMI) and fat free mass index(FFMI) in different age group. (XLS)

S2 Table The data of proportion of body composition in different age group. (XLS)

S3 Table The data of BMD value in different age group. (XLS)
